# *Photorhabdus* Species***:*** Bioluminescent Bacteria as Human Pathogens?

**DOI:** 10.3201/eid0902.020220

**Published:** 2003-02

**Authors:** John G. Gerrard, Samantha McNevin, David Alfredson, Ross Forgan-Smith, Neil Fraser

**Affiliations:** * Gold Coast Hospital, Southport, Queensland, Australia; † Queensland Medical Laboratory, West End, Queensland, Australia; ‡ Harbour City Family Practice, Gladstone, Queensland, Australia

**Keywords:** *Photorhabdus asymbiotica*, *Xenorhabdus*, insect-human infection, transgenic *Yersinia*, *Heterorhabditis*

## Abstract

We report two Australian patients with soft tissue infections due to *Photorhabdus* species. Recognized as important insect pathogens, *Photorhabdus* spp. are bioluminescent gram-negative bacilli. Bacteria belonging to the genus are emerging as a cause of both localized soft tissue and disseminated infections in humans in the United States and Australia. The source of infection in humans remains unknown.

Bioluminescence is the production of visible light by a chemical reaction in a living organism. The property is rarely reported in the clinical bacteriology laboratory because bacterial bioluminescence is seen primarily in marine species. *Photorhabdus* spp (family: *Enterobacteriaceae*) are the only terrestrial bacteria known to exhibit this property (*1*). The classification within the genus is complex with three currently recognized species: *P. luminescens*, *P. temperate*, and *P. asymbiotica* (*2*). Several subspecies are recognized.

*Photorhabdus* spp. have been the subject of intensive study by agricultural scientists because of the role these bacteria play in controlling insects. Insects, like humans, are subject to infestation by nematodes (*3*). *Photorhabdus* spp. inhabit the gut of some insect-pathogenic nematodes (*Heterorhabditis* spp.), where they form a symbiotic relationship. Nematode species of this type are able to invade the larvae of susceptible insects and release *Photorhabdus* spp. The bacteria proliferate and promote nematode reproduction by killing the insect larvae.

Insect-pathogenic nematodes harboring *Photorhabdus* spp are used as biopesticides in a number of countries, including the United States and Australia. Agricultural scientists are also attempting to develop insect-resistant transgenic crops by using insecticidal toxin genes derived from *Photorhabdus* spp (*4*).

Genes encoding homologues of insecticidal toxins from *Photorhabdus* spp occur naturally within the genome of *Yersinia pestis*, the cause of plague. Lateral transfer of genetic material between *Photorhabdus* and *Yersinia* species is thought to have resulted from their common association with insects as bacterial pathogens (*5*).

Human infection with *Photorhabdus* spp. has been described in two previous publications—six cases from the United States (*6*) and four cases from South Eastern Australia (Victoria and New South Wales) (*1*). We report two additional recent human cases of *Photorhabdus* infection from the Australian state of Queensland.

## The Study

### Patient 1

A 39-year-old male pest controller from Gladstone on a routine visit to his general practitioner in April 2001 inquired about the recent appearance of a red macule, 8 mm in diameter, on the medial aspect of his right ankle. No specific treatment was given. When he was seen again 18 days later, a painful, necrotic ulcer, about 12 mm in diameter, had developed at the original site of the red spot. An exudates was produced, although polymorphs were observed rarely, and no microorganisms were identified on Gram stain. A gram-negative organism later identified as *Photorhabdus* sp*.* was isolated in pure growth from the exudate. The patient began a 10-day course of oral cephalexin. When he was observed again 11 days later, he exhibited a persistent discharge with surrounding cellulitis. He was therefore prescribed a 10-day course of oral amoxycillin-clavulanate. Three weeks later, the ulcer appeared to be healing; after another 6 weeks, signs of infection had again developed. A gram-negative organism was isolated from the exudate but was not formally identified.

The patient was prescribed an additional 7-day course of oral cephalexin. When he was observed 3 months later, the infection had resolved. In his recent work as a pest controller, he had been spraying chemical insecticides under houses and in foreign cargo ships. He had never used insect pathogenic nematodes as a biopesticide.

### Patient 2

A 78-year-old man from the Queensland Gold Coast sought treatment in January 1999 with a 3-day history of a painful, swollen right foot. The patient had a history of polymyalgia rheumatica for which he was taking prednisone, 8 mg daily. In January 1999, after working barefoot in the garden, the man noted intense pain in his right forefoot and a very small amount of bloody discharge from the web space between his fourth and fifth toes.

The next day he was seen by his general practitioner who treated him with oral dicloxacillin. Two days later he was admitted to the hospital with increasingly severe pain with extensive redness and swelling extending to his right knee. He was noted to be afebrile with a mild neutrophil leukocytosis. He was started on a regimen of intravenous dicloxacillin and gentamicin.

Surgical debridement of the right foot was required on three occasions during the first 8 days of his admission. Pus was collected for culture on two of these occasions, and tissue was obtained during the third*.* An organism identified as *Photorhabdus* sp*.* was isolated in pure culture from each of these operative specimens. The same organism was also isolated, together with *Staphylococcus aureus,* from a superficial swab collected in the emergency department on presentation. No bacterial growth was obtained from blood cultures collected on admission.

The patient was treated with intravenous gentamicin for 2 weeks and ceftazidime for 1 week. He was discharged on a 6-week course of oral ciprofloxacin. The foot remained healed on follow-up 3 months later.

*Photorhabdus* spp. can be isolated and identified to genus level by using techniques available in most clinical bacteriology laboratories. A total of five isolates from the two patients described in the current report were examined in our laboratories with standard techniques (one from patient 1 and four from patient 2). The phenotypic characteristics that the isolates displayed were typical of the genus.

**Figure 1:**
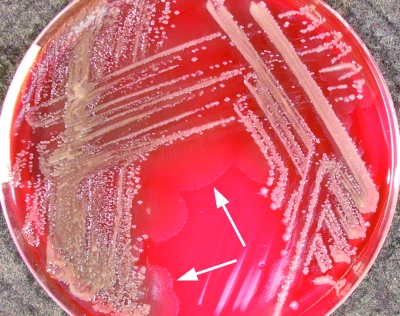
*Photorhabdus* isolate from patient 2, growing on tryptic soy agar containing 5% sheep blood, after 48 hours’ incubation at 35°C. Arrows indicate “swarming.” The colonies could be seen to glow faintly with the naked eye under conditions of total darkness after 10 minutes of adjustment.

**Figure 2:**
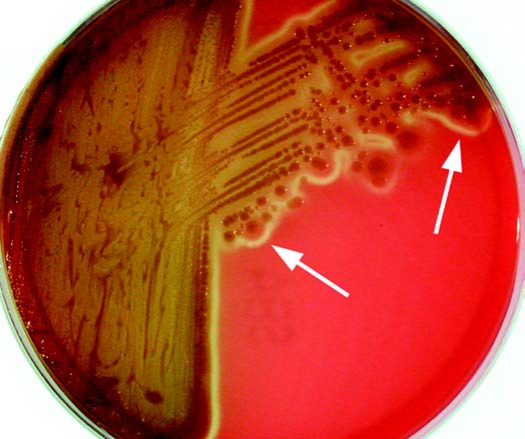
*Photorhabdus* isolate from patient 2 after 5 days growth at room temperature on sheep blood agar. Arrows indicate the characteristic thin line of “annular” hemolysis surrounding the colonies.

Colonies were formed after 24–48 hours on tryptic soy agar containing either 5% sheep or horse blood (bioMérieux, Baulkham Hills, Australia) at both 35°C and at room temperature, with a tendency to “swarm” (Figure 1). The isolates also grew on MacConkey agar. On sheep and horse blood agar, a thin line of annular hemolysis was observed 4–12 mm from the colony edge. The hemolysis was more apparent when the isolates were incubated at room temperature (Figure 2). The organisms were motile, gram-negative, rod-shaped bacteria. They were facultatively anaerobic, oxidase negative, and strongly catalase positive. Other biochemical reactions were as described previously (*1*).

The defining characteristic was the presence of faint luminescence, which could be clearly seen with the naked eye when the colonies were examined under conditions of total darkness. It was critical to this examination that the observer’s eyes be allowed to adjust to the darkness for 10 minutes.

Two commercially available automated bacterial identification systems were used in our laboratories: MicroScan Walkaway (Dade Behring Inc., MicroScan Division, West Sacramento, CA) and bioMerieux Vitek (bioMérieux; Hazelwood, MO). *Photorhabdus* spp. do not currently appear on the databases of either of these systems, which leads to misidentification (Table 1).

**Table 1:** Misidentification of *Photorhabdus* isolates from patients 1 and 2 by commercially available bacterial identification systems

System	Misidentification	Probability
MicroScan Walkaway Rapid Neg BP Combo Panel Type 4	*Shewanella putrefaciens*	99.97%
MicroScan Walkaway Neg BP Combo Panel Type 11	*Pseudomonas oryzihabitans*	85.46%
BioMérieux Vitek GNI+ V1316	*Providencia stuartii*	99%

*Photorhabdus* spp. have been shown to form a heterogeneous group based on DNA-DNA hybridization studies, 16S rDNA sequencing and polymerase chain reaction ribotyping (*2*). A polyphasic approach is now applied to classifying isolates within the genus, dividing it into three species and several subspecies. The American clinical isolates described by Farmer et al. (*6*) belong to a new species, *Photorhabdus asymbiotica* (*2*). A specific epithet has not yet been assigned to the Australian clinical isolates but they also may form a new species within the genus (*7*).

Antimicrobial sensitivity was assessed by using broth microdilution. The isolates were sensitive to a broad range of antimicrobial agents with activity against gram-negative bacteria including ciprofloxacin, gentamicin, tetracycline, ceftriaxone, and amoxycillin-clavulanate. Isolates from both patients were resistant to cephalothin and ampicillin.

## Conclusions

**Figure 3:**
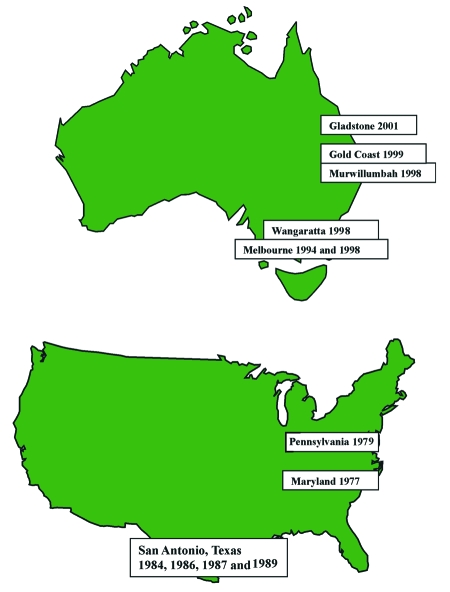
Australian and American clinical isolates of *Photorhabdus.*

Publication of information about these two cases brings to a total of 12 the number of human infections with *Photorhabdus* spp*.* documented in the medical literature (Table 2 and Figure 3). The clinical picture described in the 12 cases has generally been one of localized or more commonly multifocal skin/soft tissue infection. Such infection has had a tendency to relapse. The disseminated distribution of skin/soft tissue infection in several cases suggests hematogenous spread. Bacteremia was documented in 4/12 case-patients. Cough was documented in two of the bacteremic case-patients. In one of these, isolates of a *Photorhabdus* sp*.* were obtained from sputum as well as from blood and skin/soft tissue.

**Table 2:** Published human cases of *Photorhabdus* infection

Case no.	Year	Country	Location	Age/sex	Clinical	Alleged vector	Source of isolate
1	2001	Australia	Gladstone, Queensland	39M	Soft tissue infection right ankle (professional pest controller)		Pus from ankle ulcer
2	1999	Australia	Gold Coast, Queensland	78M	Soft tissue infection right foot		Pus and tissue from right foot
3 (1)	1998	Australia	Murwil-lumbah, New South Wales	55M	Multifocal soft tissue infections (upper and lower limbs, abdomen), pneumonia		Blood, sputum, pus and tissue
4 (1)	1998	Australia	Wangaratta, Victoria	50M	Multifocal soft tissue infections (upper and lower limbs)	Spider	Pus from soft tissue abscesses
5 (1)	1998	Australia	Melbourne, Victoria	90M	Cough and fever		Blood
6 (1)	1994	Australia	Melbourne, Victoria	11F	Multifocal soft tissue infections (lower limbs and chest)		Pus and soft tissue biopsies
7 (6)	1989	USA	San Antonio, Texas		Groin infection		Groin
8 (6)	1987	USA	San Antonio, Texas	45M	Multifocal soft tissue infection, left lower limb	Spider	Pus from lower limb abscess
9 (6)	1986	USA	San Antonio, Texas	78M	Multifocal soft tissue infection left lower limb		Pus from lower limb abscess and ulcer
10 (6)	1984	USA	San Antonio, Texas	36F	Disseminated bacterial infection		Submandible, abdomen
11 (6)	1979	USA	Pennsyl- vania	72F			Blood, skin
12 (6)	1977	USA	Maryland	80F	Endocarditis		Blood

Given the very limited clinical experience, making definitive recommendations about treatment is not possible. Antimicrobial therapy should be guided by in vitro sensitivities. The tendency for *Photorhabdus* infection to relapse suggests that prolonged therapy for a period of weeks would be prudent, perhaps with an oral fluoroquinolone.

*Photorhabdus* spp are not human commensals. The patients apparently acquired the pathogen from an unidentified source in the terrestrial environment. This hypothesis is supported by the observations that at least 4/6 of the Australian patients were engaged in outdoor activities around the time of acquisition and that the initial site of infection was on the lower limbs in more than half of Australian and American case-patients.

*Photorhabdus* spp. have never been shown to live freely in soil, although they will survive in soil under laboratory conditions (*8*). *Photorhabdus* spp. have only been isolated naturally from two nonclinical sources: insect-pathogenic nematodes (*Heterorhabditis* spp) and the insects they parasitize (beetles, moths, and the like). It seems likely therefore that *Photorhabdus* spp are transmitted to humans by a terrestrial invertebrate (nematode or arthropod), but that vector has not yet been identified.
